# Parental Self-Efficacy and Child Externalizing and Internalizing Problems during Wartime: the Indirect Effects of Parental Submission and Power Struggles

**DOI:** 10.1007/s40653-025-00806-x

**Published:** 2025-12-29

**Authors:** Muriel Balkanyi-Nehora, Merav Jedwab

**Affiliations:** 1 School of Social Work, Sapir College, Sderot, Israel; 2 The Paul Baerwald School of Social Work and Social Welfare, Hebrew University of Jerusalem, Jerusalem, Israel

**Keywords:** Parental self-efficacy (PSE), Child externalizing and internalizing problems, Parenting practices, Power struggles, Parental submission, Wartime

## Abstract

This study explored the indirect effects of negative parental practices, specifically parental submission and power struggles, in the associations between parental self-efficacy (PSE) and children’s internalizing and externalizing problems. Data were collected through an online survey conducted three months after the outbreak of the Israel-Hamas War, from 226 Israeli mothers of children aged 6–18. A parallel indirect effects model was employed to analyze the data. Findings revealed that PSE was significantly associated with children’s internalizing problems, but not with externalizing problems. Furthermore, both parental submission and power struggles showed significant indirect effects linking PSE to externalizing problems, whereas only parental submission showed an indirect effect linking PSE to internalizing problems. The practical implications of the results are discussed, emphasizing the importance of PSE as a cognitive framework shaping parenting practices. Wartime Interventions should focus on enhancing PSE and reducing negative practices, particularly parental submission and power struggles, in order to reduce child internalizing and externalizing problems.

## Introduction

On October 7, 2023, Hamas attacked Israel on an unprecedented scale, resulting in the death of over 1,300 civilians, the abduction of 240 individuals, and the displacement of over 200,000 people due to the destruction of homes and ongoing rocket attacks (Peleg & Gendelman, 2023). The brutality, including atrocities such as maiming, torture, and rape, shocked the nation and the world, with many Israelis reporting personal connections to those killed, kidnapped, or injured (Ayalon et al., 2024). Previous studies on the effect of October 7 and the ensuing Israel-Hamas War indicate a significant mental health toll, with elevated levels of posttraumatic stress disorder (PTSD), depression, and anxiety (Levi-Belz et al., 2024; Shamir-Stein et al., 2024), including among children and youth (Sabag et al., 2024; Shechory-Bitton et al., 2024).

In conflict-affected areas, children may endure life under siege and separation from family members, schools, and friends, and witness acts of torture and abuse inflicted upon loved ones (Myers-Walls, 2004; Sabag et al., 2024). Such conditions disrupt care systems and may impair family relationships, leaving family members feeling isolated, depressed, and overwhelmed by adversities (Gewirtz et al., 2008). These disruptions heighten risks to children’s cognitive, physical, and socioemotional well-being (Murphy et al., 2017; Sabag et al., 2024; Shechory-Bitton et al., 2024; Zamir et al., 2020), often manifesting as externalizing and internalizing problems (Gewirtz et al., 2008). Externalizing problems involve overactivity, aggression, poor impulse control, and tantrums; internalizing behaviors reflect emotional states such as sadness, anxiety, social withdrawal, and fearfulness (Achenbach et al., 1987; LaFreniere & Dumas, 1996).

Parents play a critical role in providing their children with emotional stability and a sense of security and normalcy. However, parenting during wartime presents unique challenges, as parents attempt to protect their children amid ongoing chaos and danger (Catani, 2018). Living under these conditions may influence parental cognitions and practices, as well as child outcomes (Eltanamly et al., 2021; Hyland et al., 2023; Nuttman-Shwartz, 2023). Previous studies have primarily focused on the direct relationship between parental cognitions and child outcomes (e.g., Kwon et al., 2023; Mak et al., 2020). However, comparatively little consideration has been given to the role of parenting practices as proximal mechanisms that may indirectly link these domains (Bodalski et al., 2023; Gewirtz et al., 2008).

An established theoretical framework in parenting research and interventions posits a structured pathway linking parental cognitions, parenting practices, and child outcomes (Bornstein et al., 2018; Powell, 2019). Parental cognitions shape how parents interpret and respond to their children’s needs, thereby influencing their parenting practices across verbal, behavioral, affective, and psychological domains (Bornstein, 2015; Holden & Smith, 2019; Johnston et al., 2018; Putnick, 2019). Longitudinal evidence, spanning toddlerhood to middle childhood, supports this framework, highlighting the mediating role of parenting practices in translating parental cognitions into child adjustment outcomes (Bornstein et al., 2018).

Guided by this theoretical perspective, the current study examines how parental cognitions and practices intersect in shaping children’s outcomes in wartime contexts, with a particular emphasis on parental self-efficacy. Parental submission and power struggles are examined as two negative parenting practices that serve as behavioral expressions of these cognitions and as mechanisms through which PSE may be indirectly associated with child outcomes. Children’s internalizing and externalizing problems are assessed as key indicators of emotional and behavioral functioning during wartime.

## Parental Cognitions in Wartime

Parenting during wartime presents significant challenges and can profoundly impact parental cognitions (Eltanamly et al., 2021). Parental cognitions are mental constructs encompassing knowledge of child development, attributions for behavior, attitudes toward the parental role, and broader parenting goals and values (Holden & Smith, 2019; Johnston et al., 2018; Putnick, 2019). They are central to how parents interpret and respond to children’s needs. Parental self-efficacy (PSE) is a central parental cognition (Bugental & Johnston, 2000; Holden & Smith, 2019) that influences parent-child relationships and child outcomes (Glatz et al., 2024). Drawing on Bandura’s (1977) concept of self-efficacy, PSE refers to parents’ belief in their ability to manage parenting challenges and positively influence their child’s development, shaping both how parents engage with their children and their persistence in the face of parenting challenges (Schuengel & Oosterman, 2019).

Stressful contexts such as war can undermine PSE (Paryente, 2021). For example, after 9/11, many parents reported helplessness and doubts about their ability to effectively protect or support their children (Beauchesne et al., 2002). In another study with refugee parents, low PSE was linked to a sense of loss, difficulties coping with new situations, and a lack of belonging (Eltanamly et al., 2021). Similarly, Israeli mothers exposed to terror attacks expressed low self-confidence, high anxiety, and worries about functioning as parents (Dekel, 2004; Kaitz et al., 2009). Additionally, children’s responses to war-related trauma can be unfamiliar to parents, further heightening uncertainty about how best to support their children (Cohen, 2009).

Research consistently links PSE with parenting behaviors. In a systematic review, Albanese et al. (2019) demonstrated that parents with high PSE were more likely to engage in proactive and nurturing behaviors, persist through challenges, and adapt to changing circumstances, thereby promoting children’s resilience, emotional well-being, and adaptive functioning. Conversely, low PSE was associated with increased parental stress and anxiety, fewer positive parenting practices, and adverse child outcomes (see also Junttila et al., 2015). Children of parents with low PSE were more likely to exhibit behavioral, social, and academic difficulties (Jones & Prinz, 2005).

### Parental Self-Efficacy and Child Outcomes

PSE is a critical factor influencing the parent-child relationship and children’s behaviors and developmental outcomes (Albanese et al., 2019; Glatz et al., 2024). Parents with lower PSE tend to have children with higher levels of internalizing (Junttila & Vauras, 2014) and externalizing problems (Fass et al., 2018; Martin et al., 2000). Further, PSE supports the maintenance of consistent routines and discipline, which are essential for children’s sense of security and behavioral regulation during wartime (Feldman & Vengrober, 2011). Although there is substantial evidence demonstrating the impact of parenting on children’s internalizing and externalizing problems (Inguglia et al., 2022; Wang et al., 2025; Zou, 2021), much less is known about the impact of PSE following traumatic events (Gewirtz et al., 2008).

## The Indirect Role of Parenting Practices

Building on the documented associations between PSE and children’s emotional and behavioral outcomes (Albanese et al., 2019; Glatz et al., 2024), researchers have emphasized the need to understand the indirect pathways through which these associations occur (Bodalski et al., 2023; Gewirtz et al., 2008). One well-supported explanation is that parenting practices serve as mediating mechanisms through which parental cognitions shape child adjustment (Bornstein et al., 2018; Patterson, 1997; Powell, 2019).

The first link in this indirect pathway concerns the relationship between PSE and parenting practices. Higher levels of PSE are consistently linked to a greater use of positive parenting practices, such as warmth, involvement, and consistent discipline, which promote children’s emotional well-being and reduce the likelihood of internalizing and externalizing problems (Gittins et al., 2020; Glatz et al., 2024; Hamovitch et al., 2019; Nicolas et al., 2019). In contrast, parents with low PSE tend to display negative practices such as inconsistent discipline, rejection, aggression (Daganzo et al., 2014), overinvolvement, and aversion (Yap et al., 2014). These patterns align with Bandura’s (1977) self-efficacy theory, which states that self-efficacy beliefs predict whether people engage in a given behavior, the effort they put forth, and their persistence in the face of obstacles (Bandura, 1977; see also Dix, 1991).

The second step in the indirect pathway is the link between parenting practices and children’s outcomes. Positive parenting practices, specifically supportive parenting and maternal warmth, have been shown to be associated with fewer externalizing and internalizing behaviors (Rothenberg et al., 2020). In contrast, negative parenting practices (e.g., rejection, criticism, harsh parenting, and indifference, or coercion; Hentges et al., 2019) increase the risk of internalizing and externalizing problems (Deater-Deckard et al., 2006; Inguglia et al., 2022). Harsh discipline, including extreme power assertion, corporal punishment, as well as behavioral and psychological aggressions, has been linked to aggressive behaviors (Liu & Wang, 2018; Yap et al., 2014), as well as internalizing problems such as depressive symptoms and cognitive difficulties (Dzeidee Schaff et al., 2024).

Wartime increases the likelihood of negative parenting practices (Cobham et al., 2016; Eltanamly et al., 2021; Gewirtz et al., 2008), which in turn are linked to children’s emotional and behavioral problems (Halevi et al., 2017). Patterson’s (1982) social interaction model describes how stressful life circumstances may shape parenting practices and child outcomes. Drawing on family observational data, Patterson identified coercion as a central mechanism underlying child behavior problems and proposed that stressful life circumstances may contribute to coercive family interactions (Patterson et al., 1984, 1989). Under such conditions, parenting practices may contribute to escalating dynamics (Lavi-Levavi et al., 2013).

## Parental Submission and Power Struggles

Based on Patterson’s coercion model, the escalation model (Omer, 2004, 2021) refers to cycles of increasingly negative interactions that may erode relationship quality and contribute to children’s behavioral problems. These processes typically encompass two types of negative parental practices: parental submission and power struggles. Submission occurs when parents consistently comply with their child’s demands or tolerate misbehavior at the expense of their own values (Omer, 2004, 2021), to avoid conflict or reduce immediate stress (Konca & Tantekin Erden, 2021). Conversely, power struggles arise when parents and children engage in conflicts in which both sides attempt to assert control (Coyne et al., 2014). Patterson and MacCoby (1980) suggest that both forms of coercive patterns, parental submission and power struggles, can escalate conflict in the parent-child relationship and reinforce negative child behaviors. While parental submission reinforces the child’s negative behaviors through concessions, power struggles fuel cycles of increasingly negative interactions that perpetuate both parents’ frustration and children’s misbehavior.

## The Current Study

While the direct associations of PSE and parenting practices with externalizing and internalizing problems are well established (e.g., Fass et al., 2018; Martin et al., 2000), relatively few studies have examined the indirect role of parenting practices linking PSE and these outcomes (e.g., Dzeidee Schaff et al., 2024), particularly within the broader framework of parental cognitions and child outcomes (e.g., Kwon et al., 2023; Mak et al., 2020). Nevertheless, this indirect pathway has been recognized as an important direction for further research (Bodalski et al., 2023), specifically as a potential mechanism underlying child adjustment following mass trauma such as war (Gewirtz et al., 2008). Accordingly, the current study uses a survey to explore how parental cognitions and practices intersect to influence children’s outcomes in a wartime context. Based on the literature, we propose the following hypotheses:

H1 PSE would be negatively associated with child internalizing and externalizing problems.

H2 Parental submission and power struggles would have parallel indirect effects on the associations between PSE and child internalizing and externalizing problems.

## Method

### Procedure

An online survey was distributed via social media (Facebook, WhatsApp) several months into the war, from January to March 2024, and administered via the Qualtrics platform. Participants filled out a sociodemographic questionnaire, followed by the study measures. Questionnaires were identified by code only, ensuring the respondents’ anonymity. All participants provided informed consent before filling out the survey. The ethics committee of the authors’ institution approved the study (Approval Number: 90124).

## Participants

A total of 539 mothers responded to the survey; 48.2% completed the full survey. Thus, the current study is based on 226 mothers who completed the full survey and met the inclusion criteria: Hebrew-speaking parents living in Israel during the war with at least one child aged 6–18. Almost all mothers (97.8%) were Jewish, 91.5% were married or in a cohabiting relationship, and 84% were employed. More than half (53.1%) described themselves as secular, while a third (33.6%) described themselves as traditional Jews. Among all participants, 19.5% reported being present at the October 7 attack, while 52.7% reported that someone close to them was murdered, kidnapped, or reported missing as a result of this attack.

**Table 1:** Participants’ sociodemographic characteristics (*N* = 226)

Variable	Min–Max	*n* (%)	M (SD)
Mother’s age	21–63		39.71 (7.16)
Ethnicity			
Jewish		221 (97.8)	
Arab		5 (2.2)	
Maternal health^a^			4.40 (8.28)
Education			
Up to bachelor’s degree		128 (56.6)	
MA & PhD		98 (43.4)	
Marital status			
Married or cohabiting		207 (91.5)	
Nonmarried		19 (8.5)	
Number of children	1–8		2.58 (1.26)
Age of focal child	6–18		8.60 (4.46)
Gender of focal child			
Male		132 (58.4)	
Female		94 (41.6)	
Monthly income^b^			3.32 (1.13)
Residential area in Israel			
North		33 (14.6)	
Center		94 (41.6)	
Jerusalem		22 (9.7)	
South		77 (34.1)	
IDP^c^			
Yes		55 (24.3)	
No		171 (75.7)	
Present at the October 7 attack			
Yes		44 (19.5)	
No		182 (80.5)	
Someone close killed, kidnapped, or reported missing^d^			
Yes		119 (52.7)	
No		107 (47.3)	
Nuclear family member currently serving in the military^e^			
Yes		73 (32.3)	
No		153 (67.7)	

^a^From 1 “very poorly” to 5 “very good”. ^b^From 1 “much below given average” to 5 “much above given average”; based on a reference household income of 21,000 NIS. ^c^Internally Displaced Persons. ^d^Nuclear and close family member, neighbor, or friend. ^e^Spouse or older child

### Measures

#### Child Outcomes

Child outcomes were assessed using the Brief Problem Monitor-Parent form (BPM-P), a short version of the Child Behavior Checklist (CBCL; Achenbach et al., 2011). The BPM-P contains 19 items that index behavioral and emotional problems in the domains of internalizing, externalizing, and attention problems among children aged 6–18. The attention problems subscale consists of six items (e.g., “Can’t concentrate, can’t pay attention for long”); the externalizing problems subscale consists of seven (e.g., “Disobedient at home”); and the internalizing problems subscale consists of six (e.g., “Feels too guilty”). In the current study, only the internalizing and externalizing subscales were scored.

Mothers were asked to rate their children’s behavior over the past 30 days (0 = not true; 1 = somewhat true; 2 = very true). The externalizing subscale ranged from 0 to 14 and the internalizing subscale from 0 to 12, with higher scores indicating more severe problems. The Hebrew version of the BPM-P was adapted from the original long version of the CBCL (Zilber, et al., 1994) and was found to have good reliability and validity in Israel (e.g., Gershy et al., 2017). The internal consistency of the BPM-P in the current study was α = 0.80 for the externalizing and α = 0.81 for the internalizing subscale.

### Parental Cognition

Parental cognition was assessed using the Parental Self-Efficacy in the Context of Political Violence and Security Threats Scale developed by Pagorek-Eshel and Dekel (2015). This scale is based on the Parental Sense of Competence Scale (PSOC; Johnston & Mash, 1989). It assesses parents’ perceived confidence in their ability to fulfill their parental roles and meet their children’s needs amid security threats. The scale comprises 12 items (e.g., “I can tell when my children are distressed due to the security situation, and I can respond effectively”; “I know precisely what actions I must take to protect my children in a security emergency, for example, when an alarm sounds”) rated on a Likert scale from 1 (“strongly disagree”) to 5 (“strongly agree”). Items 2, 6, 8, 9, and 11 are reverse-coded. A higher mean score indicates greater perceived PSE in the context of political violence and security threats. The scale demonstrated high reliability in the current study, with a Cronbach’s alpha of 0.87.

### Parenting Practices

Parenting practices were assessed using the Escalation Questionnaire (Lavi-Levavi, 2009). This measure contains 21 items that assess parental emotional and behavioral reactions according to Patterson’s theory of escalation (Omer, 2004; Patterson et al., 1984). Each item is rated on a 7-point Likert scale ranging from 0 (“not true at all”) to 6 (“completely true”). In the current study, two of the measure’s four scales were used: Power Struggles (e.g., “I want to show my child that I am the boss”) and Parental Submission (e.g., “I allow my child to do things I oppose so I could get some quiet time”). The measure was developed in Hebrew (Lavi-Levavi, 2009) and validated in several studies (e.g. Gershy et al., 2017; Lavi-Levavi et al., 2013). Cronbach’s alpha for the two scales used in the current study was 0.75.

### Sociodemographic Questionnaire

Mothers completed a sociodemographic questionnaire including questions on age, health, education, income, area of residence, marital status, number of children, and age and gender of the focal child. Mothers with more than one child were instructed to report on only one child throughout the study. In addition, mothers were asked several questions aimed at understanding the family’s exposure to the October 7 attack and ensuing war. Four yes/no questions asked whether the family was present during the attack, whether it was subsequently displaced, and whether any close family member or friend was killed, kidnapped, reported missing, or currently serving in the military (see Table 1).

### Data Analysis

The data analyses were conducted using IBM SPSS (version 24) and the PROCESS macro for SPSS (Hayes, 2018, Model 4). First, means and SDs were calculated, followed by Pearson correlations. PROCESS was then used to test parallel indirect effects, examining whether parental submission and power struggles had indirect associations with the relationships between PSE and children’s internalizing and externalizing problems in separate models. Indirect effects were considered significant if the 95% confidence interval (CI), based on 5,000 bootstrap resamples, did not include zero.

## Results

### Descriptive Statistics and Associations among Study Variables

Table 2 presents descriptive statistics and Pearson correlations among the study variables. PSE was negatively associated with parental submission, power struggles, and internalizing and externalizing problems. Parental submission and power struggles were positively related to both internalizing and externalizing problems. Among the background variables, monthly income, health, number of children, and age of the focal child were significantly correlated with all four variables and were therefore entered as covariates in subsequent analyses.

**Table 2:** Descriptive statistics and pearson correlations among the study variables (*N* = 226)

Variables	1	2	3	4	5	M (SD)
1. Internalizing problems						10.31 (3.16)
2. Externalizing problems	0.40**					12.78 (3.36)
3. PSE	− 0.25**	− 0.25**				3.56 (0.67)
4. Parental submission	0.29**	0.38**	− 0.32**			2.29 (0.71)
5. Power struggles	0.20**	0.38**	− 0.22**	0.24**		2.32 (0.69)
6. Health^a^	− 0.12	− 0.19**	0.24**	− 0.20**	− 0.04	4.40 (0.82)
7. Monthly income^b^	− 0.08	− 0.28**	0.16**	− 0.08	− 0.05	3.32 (1.13)
8. Number of children	0.21**	0.18**	0.01	0.03	0.15*	2.57 (1.26)
9. Age of focal child	0.30**	0.05	− 0.02	− 0.04	0.18**	8.60 (4.46)
10. Marital status^c^	− 0.17**	− 0.14*	− 0.02	− 0.07	− 0.03	
11. Exposure to the October 7 attack^d^	− 0.09	0.01	0.06	− 0.04	− 0.13*	
12. IDP^e^	0.21**	0.27**	− 0.06	0.09	0.12	
13. Military service^f^	− 0.04	0.09	0.08	0.06	0.03	

^a^ From 1 “very poorly” to 5 “very good”. ^b^From 1 “much below given average” to 5 “much above given average”. ^c^Married or cohabiting = 1, Nonmarried = 0; ^d^Someone close was killed, kidnapped, or reported missing: Yes = 1, No = 0; ^e^IDP= Internally Displaced Persons: Yes = 1, No = 0; ^f^Nuclear family member currently serving: Yes = 1, No = 0. *p <.05, ** *p* <.01, *** *p* <.001

### Parallel Indirect Effects Analysis

To examine H2, we tested two parallel indirect effects models. In each, PSE served as the independent variable, and parental submission and power struggles were the variables through which the indirect effects were estimated, while internalizing and externalizing problems were the dependent variables (tested in separate models). For internalizing problems, the total effect of PSE was significant (*b* = −1.07, *p* <.001). This effect included significant indirect effects through parental submission and power struggles (total indirect effect = − 0.36, CI 95% [−0.66, − 0.14]), suggesting that the association between lower PSE and higher internalizing problems was indirectly associated with these parenting practices.

**Fig. 1:**
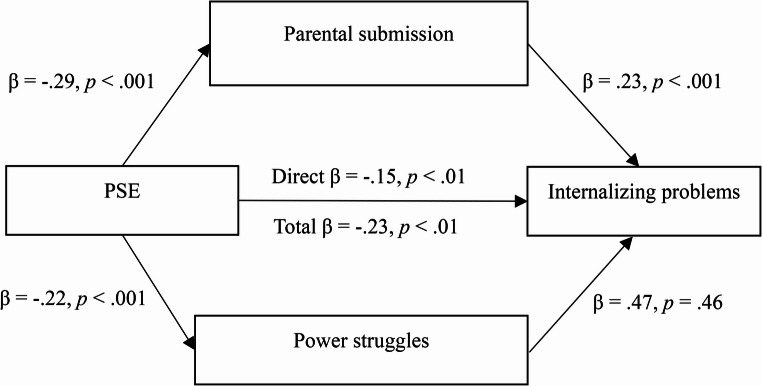
Parallel Indirect Effects of Parental Submission and Power Struggles in the Relationship Between PSE and Internalizing Problems (Standardized Regression Coefficients)

Exploring specific indirect effects showed that the relationship between PSE and children’s internalizing problems had a significant indirect effect through parental submission (*b* = −0.31, CI 95% [−0.60, − 0.09]). Specifically, lower levels of PSE were associated with higher parental submission (*b* = − 0.30, *p* <.001), which, in turn, was associated with increased internalizing problems (*b* = 1.03, *p* <.001). Similarly, the relationship between PSE and internalizing problems was tested with power struggles included in the model. However, this indirect effect was not significant (*b* = − 0.05, CI 95% [−0.22, 0.08]), indicating that the indirect effect through power struggles was nonsignificant. Although PSE was negatively associated with power struggles (*b* = − 0.23, *p* =.001), power struggles did not significantly predict internalizing problems (*b* = 0.21, *p* =.468). Finally, PSE retained a direct relationship with internalizing problems, even after accounting for the mediators (*b* = − 0.71, *p* =.023), indicating a partial indirect effect. In other words, the association between PSE and children’s internalizing problems was only partially explained through parental submission and power struggles. The standardized indirect effects model is presented in Fig. 1.

**Fig. 2:**
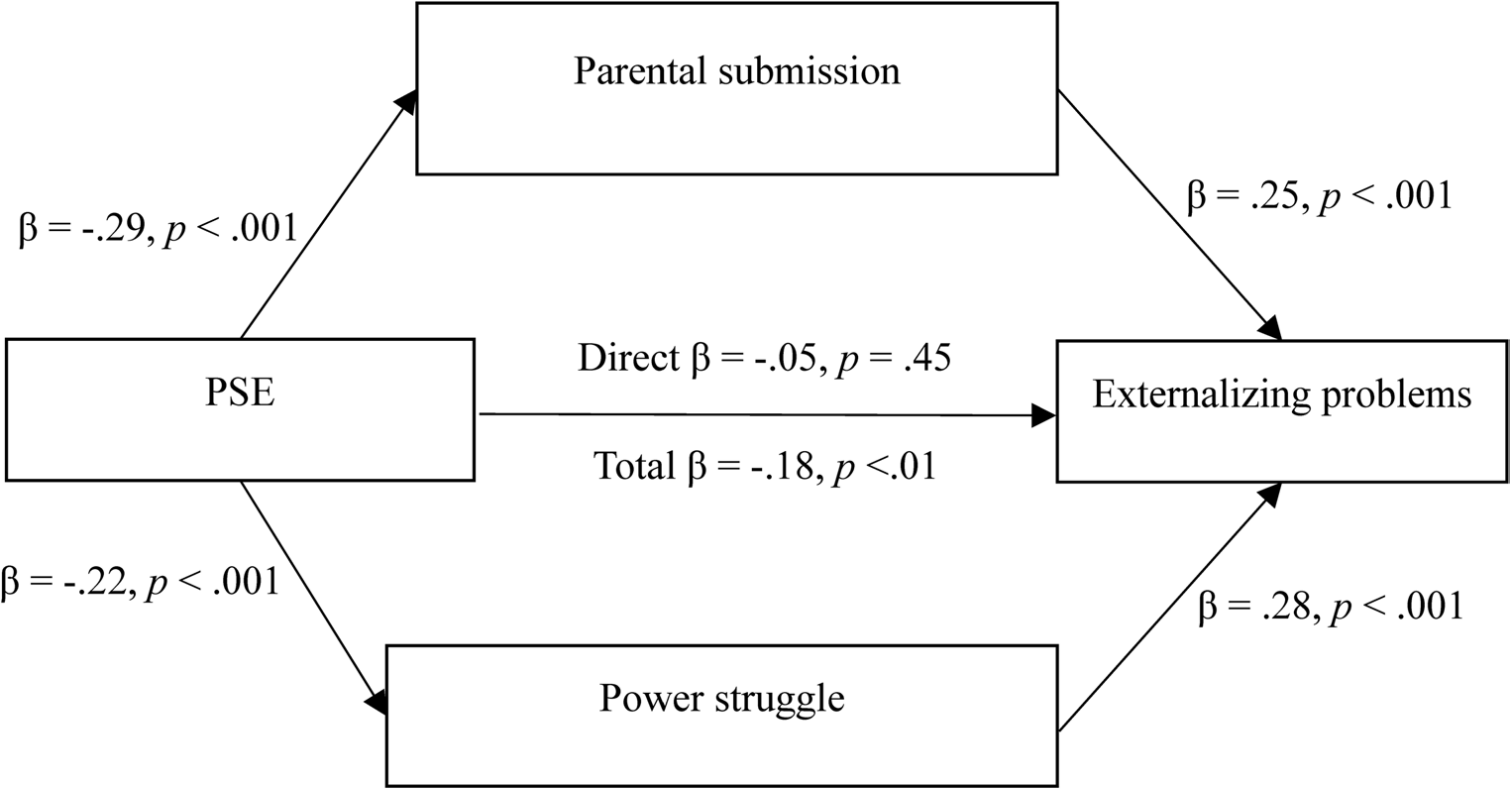
Parallel Indirect Effects of Parental Submission and Power Struggles in the Relationship Between PSE and Externalizing Problems (Standardized Regression Coefficients)

A different pattern emerged for externalizing problems. The total effect of PSE on externalizing problems was significant (*b* = −0.89, *p* =.005). This effect included significant indirect effects through parental submission and power struggles (Total indirect effect = − 0.66), CI 95% [−0.99, − 0.37]). The relationship between PSE and externalizing problems showed a significant indirect effect through parental submission (*b* = − 0.35), CI 95% [−0.60, − 0.14]). Specifically, lower levels of PSE were associated with higher parental submission (*b* = − 0.30, *p* <.001), which, in turn, was associated with increased externalizing problems (*b* = 1.17, *p* <.001). The relationship between PSE and externalizing problems also showed a significant indirect effect through power struggles (*b* = −0.31, CI 95% [−0.59, − 0.10]). Lower PSE was associated with higher power struggles (*b* = − 0.23, *p* =.001), which was also significantly associated with increased externalizing problems (*b* = 1.37, *p* <.001). Finally, PSE did not retain a direct association with externalizing problems after accounting for the indirect effects (*b* = − 0.23, *p* =.451), indicating a full indirect effect. That is, the association between PSE and children’s externalizing problems was fully explained through the combined indirect effects of parental submission and power struggles. The standardized indirect effects model is presented in Fig. 2.

## Discussion

This study explored the relationships between parental cognitions, parenting practices, and child outcomes in the context of wartime. Specifically, it examined parental submission and power struggles as parallel indirect pathways linking PSE with child internalizing and externalizing problems. The findings provide partial support for H1: while a significant direct association was found between lower PSE and increased internalizing problems, no significant direct relation emerged between PSE and externalizing problems. This suggests that parents’ diminished confidence in managing parenting challenges may be associated with greater internalizing difficulties in children, consistent with prior research (Fass et al., 2018; Martin et al., 2000).

In contrast, and inconsistent with previous findings (e.g., Glatz et al., 2024), there was no significant relation between PSE and child externalizing problems. This may reflect the unique wartime context and the specific focus on parents’ perceived confidence in effectively fulfilling their parenting roles and meeting their children’s needs (Pagorek-Eshel & Dekel, 2015). PSE may operate differently under acute stress and threat conditions than in everyday contexts. As PSE is a complex construct of parental cognition that may shift depending on situational demands such as war (Eltanamly et al., 2021; Pagorek-Eshel & Dekel, 2015), PSE in the context of political violence may represent an event-dependent cognition (Bugental & Johnston, 2000) rather than one tied to routine parenting (Johnston et al., 2018). From this standpoint, biopsychosocial factors such as age (Bodalski et al., 2023) and developmental period (Glatz & Buchanan, 2015) may moderate the link between PSE and externalizing problems, weakening it to the point of non-significance. This suggests that the relationship between parental cognitions and child behavior may vary across developmental stages or family contexts. Further research should examine how parent, child, or family factors moderate wartime PSE’s associations with children’s externalizing behavior.

Beyond direct associations, the findings confirm the indirect effects of parental submission and power struggles in the relationship between PSE and child outcomes. The association between PSE and externalizing problems showed indirect effects through both parental submission and power struggles. This is in accordance with Patterson and MacCoby’s (1980) suggestion that both parental submission and power struggles escalate conflict in the parent-child relationship and are linked to children’s negative behaviors. However, for externalizing problems, parental submission and power struggles operate in parallel and mutually reinforce each other (Omer, 2004, 2021; Patterson, 1982; Patterson et al., 1984), whereas the association between PSE and children’s internalizing problems showed an indirect link only through parental submission.

During wartime, parents’ confidence and ability to effectively fulfill parenting roles may be compromised (Cobham et al., 2016; Eltanamly et al., 2021). Impaired PSE may be associated with escalation cycles in parent-child relationships, leading to an increase in negative parenting practices and child behavioral difficulties (Daganzo et al., 2014; Omer, 2004; Patterson, 1982; Patterson et al., 1984). This aligns with Bandura’s (1977) theory, which states that self-efficacy beliefs predict behavior, and with Dix’s (1991) behavioral theory, which posits that parental cognitions, such as PSE, influence parenting practices. These practices, in turn, are positively associated with children’s reactions and behaviors (Cobham et al., 2016; Halevi et al., 2017).

The current study highlights parental submission as a key indirect pathway linking PSE and both internalizing and externalizing problems. Parental submission reflects parents’ tendency to yield to children’s demands without appropriate boundaries. It is also related to poor parental monitoring, which often increases the child’s demands and harsher behavior (Lavi-Levavi et al., 2013; Patterson, 1982). This practice aims to avoid conflict or reduce immediate stress (Baumrind, 1991). Children in these situations may fail to learn how to regulate their emotions, manage frustration, or respect boundaries, resulting in both internalizing (Fass et al., 2018; Pinquart, 2016) and externalizing problems (Patterson et al., 1989).

Furthermore, the analysis revealed that power struggles showed significant indirect effects only in the association between PSE and children’s externalizing problems. These struggles exacerbate externalizing behaviors by creating confrontational dynamics that may reinforce defiance and opposition in children (Lavi-Levavi et al., 2013). As a parental practice, they reflect escalating cycles of control and resistance, where parents increase demands or control in response to children’s resistance, and children escalate their oppositional behavior in turn (Hoffman, 2013). Parents with low PSE, especially in wartime, may lack confidence and capacity to discipline, fostering a conflictual environment that undermines the parent-child relationship and supportive interactions. Such conflict can lead children to model aggressive or defiant behavior as a learned response to stress or perceived threats to autonomy (Eltanamly et al., 2021; Fass et al., 2018). Power struggles may also result in inconsistent or harsh disciplinary practices, further exacerbating externalizing problems (Glatz & Buchanan, 2015). In this context, wartime stress may not only undermine PSE (Eltanamly et al., 2021) but also increase parental stress (Conway et al., 2013). Although related, PSE and parental stress are conceptually and empirically distinct constructs (Crnic & Ross, 2017). Future research should explore their separate yet intertwined roles in escalating parenting difficulties and negative outcomes in children.

Finally, the findings align with previous studies demonstrating that negative parenting practices show indirect effects in the associations between PSE and externalizing problems (Fass et al., 2018; Glatz & Buchanan, 2015). Although earlier research did not specifically address parental submission and power struggles, it identified indirect pathways through negative parenting practices, such as punitive discipline, in this relationship. Parenting practices are multidimensional, encompassing verbal, physical, affective, and psychological behaviors (Bornstein, 2015; Putnick, 2019). The present study extends this body of work by highlighting parental submission and power struggles as negative parenting practices that show indirect associations between PSE and both child externalizing and internalizing problems. The impact may be especially pronounced in war-affected families, possibly by exacerbating the direct effect of war on children’s behavior (Eltanamly et al., 2021). In line with Patterson (1982; Patterson et al., 1984; Patterson & MacCoby, 1980), and Omer’s escalation model (2004, 2021), our findings highlight parental submission and power struggles as two coercive practices that reflect boundary dissolution (Thompson et al., 2024). Submission reinforces negative behaviors through concession, whereas power struggles escalate hostility, perpetuating cycles of conflict and aggression through parents’ attempts to impose authority or respond to children’s aggression. Particularly under stressful circumstances, such as those found in wartime (Cobham et al., 2016; Eltanamly et al., 2021), parents may be even more likely to submit to their child’s demands and engage in power struggles, both of which contribute to the worsening of the child’s behavior. However, despite theoretical and empirical support for the links between coercive patterns and children’s externalizing problems (e.g., Brumariu et al., 2018), research examining them is limited and relatively inconsistent. Further research is needed to clarify how coercive dynamics contribute to internalizing problems (Thompson et al., 2024).

### Implications for Practice

Our findings highlight the importance of interventions that strengthen parent-child relationships, which are indirectly associated with lower boundary violations and reduced risk of internalizing and externalizing problems. Establishing parental authority, clear boundaries, and emotional support may foster stability, resilience, and healthier behavioral outcomes (McClelland et al., 2013). Such practices are especially critical for supporting family dynamics in wartime (Eltanamly et al., 2021).

Our findings also point to the need to address parental cognitions and practices as part of wartime interventions. Enhancing parental self-efficacy offers a tangible and modifiable entry point that is linked to more adaptive parenting practices and better child adjustment. By strengthening PSE, programs may help parents minimize coercive patterns such as submission and power struggles, which are associated with higher risks of internalizing and externalizing problems. Effective wartime interventions may benefit from focusing on key parental skills, emotional regulation, impulse control, conflict resolution, and the ability to interpret and respond to a child’s cues appropriately, which foster supportive parenting (Murphy et al., 2017). This approach aligns with trauma-informed care frameworks and offers practical pathways for clinicians working with war-affected families.

Increasing PSE during wartime can be achieved through emotional and practical support, as well as tools for effective parenting under stress (Pagorek-Eshel & Dekel, 2015). Stress-management programs, such as mindfulness or resilience training, can help parents regulate their emotions and build confidence in handling challenges (Burgdorf et al., 2019; Eltanamly et al., 2021). Positive parenting practices are essential for children’s behavioral regulation and overall development (McClelland et al., 2013).

To counter negative parental practices, interventions can encourage parental presence without escalation. Nonviolent resistance (NVR), developed by Omer (2001, 2004), trains parents to cope with children exhibiting violent behavior and other discipline-related issues. Evaluations show that NVR reduces parental helplessness (Lavi-Levavi et al., 2013; Lebowitz et al., 2012). This training can be particularly effective if implemented in cases of escalation processes that reflect coercive patterns in parent-child relationships, particularly during wartime.

#### Limitations, Strengths, and Future Directions

The study was conducted in Israel during wartime, without prewar data on PSE. This absence of baseline data complicates our ability to assess how the war context influenced PSE, parenting practices, and child outcomes over time. Future research should incorporate longitudinal data. Secondly, the relatively small sample size limits generalizability to broader populations, despite the fact that the war impacted parents and children on both sides of the conflict (Paltiel et al., 2024). Future research should also examine societies in comparable contexts. Thirdly, fathers were not included due to their typically low participation in online surveys, which limits our understanding of the relationship between paternal PSE and child outcomes. Future studies should aim to engage fathers to explore potential gender differences in PSE and parenting practices (Dzeidee Schaff et al., 2024). Fourthly, reliance on self-reported measures of PSE and parenting practices may have introduced response bias, as parents might overestimate their capabilities (Jones & Prinz, 2005). Fifthly, the study did not consider non-war-related contextual factors and child-related variables (e.g., economic hardship, parental physical and mental health, prior exposure to trauma, coparenting, and child temperament), which can significantly affect both PSE and child outcomes. These factors should be incorporated in future longitudinal designs to provide a more comprehensive understanding of the mechanisms linking parental cognitions, parenting practices, and child adjustment.

Finally, parental cognitions, practices, and children’s emotional and behavioral problems are bidirectional and transactional (Jones & Prinz, 2005). These associations strengthen over time unless disrupted by extraneous benign factors (Schuengel & Oosterman, 2019). However, the cross-sectional study design prevents disentangling the bidirectionality of PSE, parenting practices, and children’s internalizing and externalizing problems. Longitudinal studies could clarify the bidirectionality of these transactional pathways, test the conclusions of the current study, and provide a stronger foundation for professional interventions.

In addition to its limitations, the current study offers several strengths. First, it was conducted during wartime in Israel, providing timely insights into parental cognitions, parenting practices, and child outcomes in a context where empirical evidence is scarce. Second, the study identifies both PSE and specific negative parenting practices (submission and power struggles) as factors indirectly associated with children’s emotional and behavioral outcomes, highlighting concrete, modifiable targets for intervention. Moreover, the study effectively translates theoretical insights into practical recommendations for wartime intervention programs, emphasizing parental confidence, emotional regulation, and non-coercive strategies such as Nonviolent Resistance (NVR).

## Conclusion

This study provides important insights into the mechanisms through which parental cognitions affect child outcomes during wartime. Our findings demonstrate that PSE, as a critical parental cognition, shapes negative parenting practices, specifically parental submission and power struggles. Lower PSE was associated with greater engagement in both behaviors.

The data revealed distinct indirect pathways: both parental submission and power struggles indirectly linked PSE with children’s externalizing problems. This suggests that when parents feel less efficacious during conflict, they are more likely to alternate between yielding to children’s demands and engaging in conflictual dynamics, both of which contribute to elevated externalizing symptoms. For internalizing problems, parental submission alone emerged as a significant indirect pathway, suggesting that passivity and withdrawal may be particularly detrimental to children’s emotional well-being.

These findings underscore the need to address both cognitive and practical dimensions of parenting. Interventions that enhance parental self-efficacy and provide concrete parenting strategies should be prioritized. Strengthening parents’ confidence in their caregiving abilities, alongside support in reducing both submissive and conflictual parenting practices, may help mitigate the negative impact of wartime on children’s emotional and behavioral functioning.

## Data Availability

The datasets generated and/or analyzed during the current study are available from the corresponding author upon reasonable request after publication.
